# Fully Convolutional Neural Network for Vehicle Speed and Emergency-Brake Prediction

**DOI:** 10.3390/s24010212

**Published:** 2023-12-29

**Authors:** Razvan Itu, Radu Danescu

**Affiliations:** Computer Science Department, Technical University of Cluj-Napoca, St. Memorandumului 28, 400114 Cluj-Napoca, Romania; radu.danescu@cs.utcluj.ro

**Keywords:** computer vision, scene perception, monocular vision, vehicle state estimation, CNN

## Abstract

Ego-vehicle state prediction represents a complex and challenging problem for self-driving and autonomous vehicles. Sensorial information and on-board cameras are used in perception-based solutions in order to understand the state of the vehicle and the surrounding traffic conditions. Monocular camera-based methods are becoming increasingly popular for driver assistance, with precise predictions of vehicle speed and emergency braking being important for road safety enhancement, especially in the prevention of speed-related accidents. In this research paper, we introduce the implementation of a convolutional neural network (CNN) model tailored for the prediction of vehicle velocity, braking events, and emergency braking, employing sequential image sequences and velocity data as inputs. The CNN model is trained on a dataset featuring sequences of 20 consecutive images and corresponding velocity values, all obtained from a moving vehicle navigating through road-traffic scenarios. The model’s primary objective is to predict the current vehicle speed, braking actions, and the occurrence of an emergency-brake situation using the information encoded in the preceding 20 frames. We subject our proposed model to an evaluation on a dataset using regression and classification metrics, and comparative analysis with existing published work based on recurrent neural networks (RNNs). Through our efforts to improve the prediction accuracy for velocity, braking behavior, and emergency-brake events, we make a substantial contribution to improving road safety and offer valuable insights for the development of perception-based techniques in the field of autonomous vehicles.

## 1. Introduction

In the pursuit of making autonomous vehicles a commonplace reality, the fusion of computer vision methods and additional measurement data, particularly within the realm of perception, represents a substantial research interest. The ability to acquire and process accurate measurements is fundamental in the context of self-driving vehicles, where the integrity of decision-making processes relies on the reliability of sensory information. While multiple sensor methods have been employed to achieve this objective, the integration of camera video data stands out as a cost-effective and easily deployable solution. This paper underscores the significance of making use of camera video data alongside complementary sensorial measurements, proving its potential to enhance road safety and driving autonomy.

The rationale behind the widespread adoption of camera video data is multifaceted. Not only is it a more economical option in comparison to sophisticated sensors like LIDAR (light detection and ranging) or radar, but it is also simpler to integrate into the existing infrastructure. These advantages translate into a higher potential for the development of self-driving technologies across diverse vehicle fleets, regardless of their price point or sophistication. Furthermore, cameras provide a rich source of visual information, making them an ideal candidate for understanding complex road scenes and driving scenarios. Perception-based strategies for complex autonomous actions, such as driving, represent an on-going challenge in the field of artificial vision and learning. Recent methods for visual perception based on deep-learning techniques are being used to evaluate their robustness and efficacy for understanding driver intentions and behavior, ultimately improving road safety. Robust vehicle speed analysis and prediction is essential for ego-vehicle state estimation for autonomous driving and driver assistance systems. According to the National Highway Traffic Safety Administration (NHTSA), human error remains the primary contributing factor to road accidents, and speeding is identified as a significant issue leading to fatal accidents [1]. The same fact is indicated by Transport Canada as well, in [2].

In this context, this research paper presents a novel approach that capitalizes on sequences of continuous 20 images captured from the forward road scene. The utilization of convolutional neural networks (CNNs) for image processing and classification [3] has gained widespread popularity in the research community. Therefore, by leveraging a straightforward CNN architecture, this approach not only generates real-time emergency-brake signals but also accurately predicts the current vehicle velocity. By fusing information from both image data and additional sensor measurements, this method showcases the potential for increased road safety by affording self-driving vehicles the capability to respond to hazardous situations promptly and effectively, acting as a forward collision-warning system (FCW).

Beyond its immediate applications, the significance of this research extends into the domain of video forensics and accident analysis. The detailed road-scene data captured by cameras can serve as invaluable resources for post-incident investigations, facilitating a better understanding of events leading to accidents. Thus, this paper not only contributes to the advancement of self-driving technologies, but also holds promise for enhancing road safety analysis and incident reconstruction through the comprehensive analysis of video data. In doing so, it paves the way for a future where self-driving vehicles, armed with the fusion of computer vision and precise measurement data, play an indispensable role in redefining the landscape of road safety and driving experiences.

The main contributions of this work are the following:

1. An end-to-end lightweight CNN model to predict vehicle velocity, brake, and emergency-brake signals from a sequence of images.
2. A method to generate emergency-brake signals from accelerator and brake-pedal pressure data obtained from the datasets.
3. An automated solution for generating the training, testing, and validation datasets and for training the model efficiently on reduced hardware resources and by using publicly available datasets.

## 2. Related Work

In the context of enhancing road-traffic safety, the detection of hazardous situations remains essential. In this section, we conduct a review of existing work in the domain of self-driving vehicles. Some published methodologies leverage sensorial input, while others rely only on imagery data as input sources. There are also strategies and solutions that combine multi-sensorial input to predict hazardous situations or driver intentions within driving scenarios. The related work in this section focuses mainly on methodologies that incorporate both visual input (images captured from the scene) and sensorial input or measurements. We will first present work that features only image data as input. Early studies explored the detection of brake lights from vehicles using image data as input. For instance, Ref. [4] applied an image-processing algorithm in the HSV color space to extract vehicle tail lights, with the aim of identifying instances where the leading vehicle is braking, potentially requiring emergency-braking interventions.

### 2.1. Methods Using Only Image-Data

Several studies have delved into predicting emergency-braking scenarios using CNNs. Ref. [5] focused on brake-light detection through a vision-based CNN approach, employing a deep CNN architecture based on the Yolo detector [6] to accurately identify brake-light signals. This contributes significantly to the prediction of emergency-braking situations. 

Similarly, Ref. [7] proposed a vision-based method for predicting emergency braking, integrating a CNN and LSTM [8]. Their approach concentrated on processing sequential images from road-traffic tunnels, showcasing the efficacy of deep learning in anticipating emergency-braking events.

Further expanding on this theme, Ref. [9] introduced six end-to-end deep-learning architectures for directly generating driving actions, such as predicting vehicle speed and steering angle. The authors made use of CNNs and recurrent neural networks (RNNs), particularly gated recurrent units, with the option of using image-based or multi-sensorial data as input. Their findings indicated that the inclusion of additional data, such as velocity, can enhance the robustness and precision of results. Therefore, we will present solutions with multiple input data sources in the next section.

### 2.2. Methods Using Additional Sensorial Input

Moreover, Ref. [10] harnessed sensorial data to predict emergency-braking distances, utilizing three-dimensional accelerometer data alongside corresponding braking distances to train a neural network for distance prediction.

Incorporating multi-sensor fusion and deep-learning techniques, Ref. [11] introduced a forward collision-warning model based on a fully connected neural network. This approach was trained using various data inputs, including velocity, acceleration, and separation distance from leading objects, along with radar-based data.

In another approach, Ref. [12] developed an end-to-end deep neural network that simultaneously performed pixel-wise semantic segmentation for scene understanding and generated vehicle control commands, including steering angle and speed. Meanwhile, Ref. [13] proposed predicting longitudinal and lateral control values using LIDAR and camera fusion, relying on CNN architectures based on Inception [14] and ResNet [15].

In the realm of pedestrian prediction, Ref. [16] introduces a novel approach that uses convolutional neural networks (CNNs) and long short-term memory (LSTM) networks to predict pedestrian positions within video sequences collected from static cameras. This method leverages an occupancy grid, along with image inputs and observed trajectory data, to make predictions about forthcoming pedestrian positions within the scene.

Turning our attention to vehicle behavior prediction, Ref. [17] proposes a method that utilizes a fully convolutional network (FCN) for road-scene image extraction, combined with an LSTM network to encode the ego-vehicle’s sensorial data, including speed and angular velocity. The primary objective here is to predict the next action that the ego-vehicle should undertake.

In a similar vein, Ref. [18] presents an approach that predicts driver actions by combining scene images and sensorial data. This network takes as input images from the road-traffic scene and various measurements, including ego-vehicle speed, steering angle, and acceleration, to predict recommended driving actions.

Furthermore, Ref. [19] introduces an LSTM-based solution for predicting future driver behavior. This approach employs a CNN to represent image frames and a recurrent neural network (LSTM) to encode twelve features extracted from these frames, encompassing elements such as ego-vehicle velocity, distances, front-car data, and more. The model predicts future ego-vehicle acceleration values across multiple axes, incorporating a pretrained ResNet for feature extraction.

Addressing the specific domain of speed-control prediction, Ref. [20] utilizes positional data from the scene, processed through a CNN and LSTM, to predict ego-vehicle speed. This method incorporates Mask R-CNN [21] and graph-based techniques to capture object information and spatial relations within the scene, eventually leading to predictions of ego-vehicle speed control.

Collectively, the studies surveyed underscore the effectiveness of deep-learning techniques and image and sensorial data-processing algorithms for predicting the driver behavior and vehicle state within road-traffic scenarios.

Most of the related work highlighted here combines imagery data with sensorial data to extract current or future predictions, frequently relying on recurrent neural networks, primarily LSTM networks. However, these methods often use complex deep-learning architectures that demand significant training data and preprocessing efforts, posing challenges when aiming for integration into hardware-constrained vehicle systems, whereas our proposal focuses on utilizing raw-pixel data from a forward-facing camera, coupled with vehicular velocity expressed directly in km/h as unprocessed inputs to a CNN. This approach enables us to extract velocity, brake necessity, and emergency-brake signals from complex road-traffic scenarios in an end-to-end manner.

The use of pixel data for feature extraction in an end-to-end manner has been extensively studied, as evidenced by [22], which predicts the steering angle based on a road-traffic scene image (single-image input and one single output). In our research, we take inspiration from this approach to feature a sequence of images and augment it by integrating vehicle velocity as an additional input, thereby improving our ability to anticipate hazardous situations and enhancing the overall robustness of our methodology. Our method is based on a sequence of the previous “N” frames, with N set to 20 in our experiments, representing the image-input sequence and the velocity-measurement data. To evaluate our methodology, we employ a publicly available large-scale video dataset, which, we demonstrate, can be further extended to integrate various input data sources, as showcased in the results section of this paper. Nevertheless, our proposed approach offers a streamlined alternative by exclusively utilizing imagery data, which simplifies the process while maintaining predictive accuracy.

## 3. Solution Overview

In this research paper, we propose a convolutional neural network (CNN) model that features multiple output channels. We first designed a model solely for predicting the speed of the ego-vehicle, and then extended its capability to provide predictions for not only the vehicle speed, but also the brake-pedal pressure and emergency-brake signals.

The foundational architecture of the initial model is based on the utilization of pairs of images in conjunction with the corresponding ego-vehicle speeds, where each input image is associated with a specific velocity value, expressed in kilometers per hour (km/h). Specifically, the input dataset consists of a sequence of 20 consecutive frames, each comprising images and their corresponding velocity values. The CNN’s primary function in this initial configuration is to estimate the current vehicle speed for the given frame.

Our initial training effort focused on teaching the network to predict vehicle velocity using only the information derived from these 20 consecutive road-scene images. Encouragingly, our results demonstrated the network’s ability to effectively transform raw-pixel data from multiple frames into meaningful velocity values.

Building upon this, our subsequent objective is to expand the network’s capabilities to predict not only vehicle speed but also brake-pedal pressure and the occurrence of an emergency-brake signal. This necessitates changing the output layer to accommodate these two additional signals.

### 3.1. End-to-End Convolutional Neural Network Model

**Figure 1:**
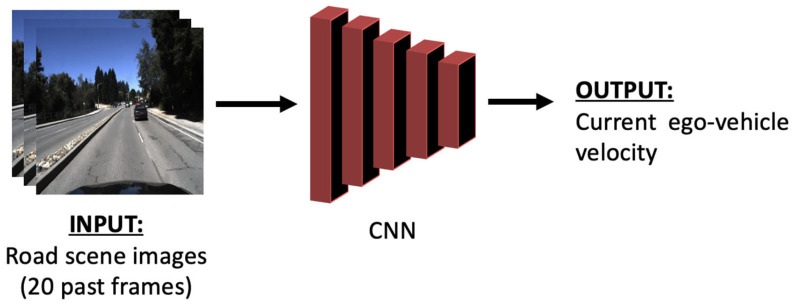
The end-to-end model that predicts velocity directly from the image data.

The initial CNN model is able to predict vehicle velocity directly from a sequence of consecutive images. The CNN architecture features convolutional layers, followed by a fully connected layer. This enables the model to predict the velocity value directly from raw-pixel data. The structure of the model is presented in Figure 1.

The model makes use of an input that consists of 20 images with the size of 300 × 300 pixels. The relevant spatial features from these images are extracted using multiple convolutional layers with various filter sizes and strides. To introduce non-linearity and enhance the model’s capacity to extract and learn intricate patterns, rectified linear unit (ReLU [23]) activation functions are utilized. To address overfitting concerns, a dropout layer is also integrated. The output of these layers is then flattened and fed into multiple fully connected layers. The final layer represents a linear-activation function that will output the predicted ego-vehicle velocity value. With approximately 5.9 million trainable parameters, this model is both easily trainable and readily deployable on alternative hardware platforms or even mobile devices.

The proposed CNN model architecture is presented as follows:

- The CNN model follows a sequential layer-by-layer structure.
- The input shape for the model is (img. height, img. width, nb. frames), which is (300, 300, 20) in this case.

Convolutional Layers:

- The first layer is a Conv2D layer with 24 filters of size (5, 5) and a stride of (2, 2). ReLU activation is applied.
- The second layer is a Conv2D layer with 36 filters of size (5, 5) and a stride of (2, 2). ReLU activation is applied.
- The third layer is a Conv2D layer with 48 filters of size (5, 5) and a stride of (2, 2). ReLU activation is applied.
- The fourth and fifth layers are Conv2D layers with 64 filters of size (3, 3). ReLU activation is applied to both layers.

Dropout Layer:

- A dropout layer with a rate of 0.5 is added to mitigate overfitting.

Fully Connected Layers:

- The output from the convolutional layers is flattened.
- The flattened output is fed into a stack of fully connected layers.
- The first fully connected layer has 100 units with ReLU activation.
- The second fully connected layer has 50 units with ReLU activation.
- The third fully connected layer has 10 units with ReLU activation.

Output Layer:

- The final layer is a dense layer with one unit and a linear activation function, providing the predicted vehicle speed as the output.

### 3.2. Extended Multiple Output End-to-End CNN Model

**Figure 2:**
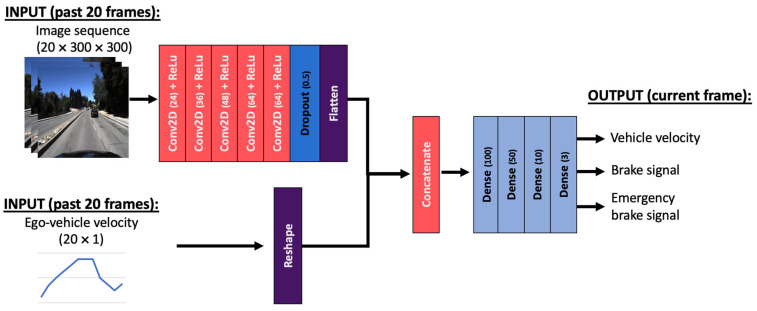
The modified CNN model with additional velocity input and additional prediction outputs.

The network is extended to include an additional input: the 1D signal for the ego-vehicle velocity, representing the measurement input. Therefore, the modified network will accept the 20 images of the road scene and 20 velocity values. The flattened output after the convolutional layers from the original 20 image input is then concatenated with the velocity-measurement data that is reshaped to maintain the same dimensionality and order. This is then followed by the same four dense layers from the initial model. The model’s output must account for the additional predicted data, the brake-pedal pressure and the emergency-brake signal, meaning that the last dense layer of the extended model will have three units (instead of one). The structure of this network is presented in Figure 2.

### 3.3. Dataset

There are many publicly available datasets for the development of vision-based self-driving vehicles, but few of them feature continuous image sequences (or videos) along with additional measurement data (such as heading, vehicle speed, brake sensor data, etc.). This is the main reason why we decided to make use of the Honda Deep Drive (HDD) [24] dataset that is available for research purposes.

The HDD dataset features approximately 104 h of continuous videos and driving data from the United States (San Francisco area) in road-traffic scenes and scenarios. The additional meta-data from the set consists of annotations regarding the driver behavior that surpass the limited data from other databases (that usually rely on simple indications like turn left, right, go straight for behavior or vehicle state estimation). The HDD dataset features sensorial information acquired from the Controller Area Network bus (CAN bus), such as ego-vehicle speed, accelerator, and brake-pedal pressure, making it fitting for evaluating how drivers behave when they engage with other road-traffic participants. The HDD dataset includes additional measurement data from the following sensors: camera, LIDAR (light detection and ranging), Global Positioning System (GPS), Inertial Measurement Unit (IMU), and CAN bus signals. The sensors are used for road-scene perception and to capture the ego-vehicle surroundings. The data obtained from the camera sensor is offered as 1280 × 720 pixels color images, at a 30 Hz frequency (frame rate). We performed an analysis of the HDD data and we found that some of the trips have missing images and associated data; therefore, not all of the dataset is at a constant 30 fps. From the total of the one hundred and thirty-seven trips, we excluded seven trips from the training and validation process when deploying the training part of the neural network. These are reserved exclusively for the evaluation part, which is described in Section 4.

From the HDD dataset trips, we systematically extracted sequences with 20 consecutive images. To ensure smooth continuity and proper coverage, a sliding window methodology was used, with a window size of twenty frames and an overlap of five frames. As a result, we generated an extensive dataset consisting of over ~57,000 continuous sequences, each composed of 20 images, along with their corresponding ego-vehicle speed and brake sensor data.

In order to address the issue of data imbalance, we filtered the generated data. Initially, we observed that roughly ~12,500 entries featured velocity values exceeding 0 km/h, while the remaining ~44,500 sequences recorded velocity data of 0 km/h. To fix this, we applied a data-balancing strategy, achieving a 50/50 distribution between stationary (velocity = 0 km/h) and moving (velocity > 0 km/h) instances. This process resulted in a refined dataset comprising of approximately ~25,000 sequences. Finally, we partitioned this balanced dataset into training and testing/validation sets, adopting an 80/20 split for further analysis and experimentation.

### 3.4. Data Analysis and Preparation

While extracting the pairs of images with corresponding velocity and brake-pedal pressure values, we opted to further examine the relation between the ego-vehicle velocity, acceleration, and brake signals in order to identify and better understand potentially hazardous situations. We decided to identify hard-brake situations by analyzing the dataset, and to generate an additional emergency-brake signal, which holds practical significance in potentially dangerous traffic scenarios. This signal is produced a few frames prior to the detection of an actual hard-braking event.

**Figure 3:**
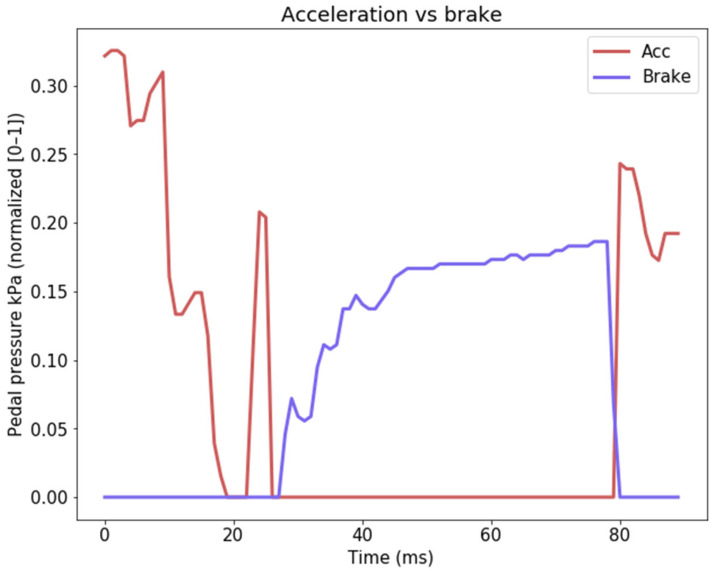
Accelerator vs. brake-pedal pressure. All values are normalized in the [0, 1] interval.

The emergency-brake signal we propose is derived from the pre-existing brake-pedal sensor data. Our analysis focuses on the first-order derivative of the brake-pedal data, which is measured in kPa within the dataset, ranging from 0 (indicating no brake application) to approximately ~7300 (representing full brake-pedal pressure). The accelerator pedal data available in the dataset falls within the range of 0 to 100, where 100 signifies a full pedal press and 0 indicates no acceleration pedal press. To facilitate consistent comparisons, we normalize these values to fit within the [0, 1] range. A visual representation of the data is provided in Figure 3 for reference.

Acceleration and brake-pedal inputs are rarely concurrent, allowing us to confidently utilize either sensor to anticipate hazardous scenarios necessitating an impending stop (hard-braking event).

**Figure 4:**
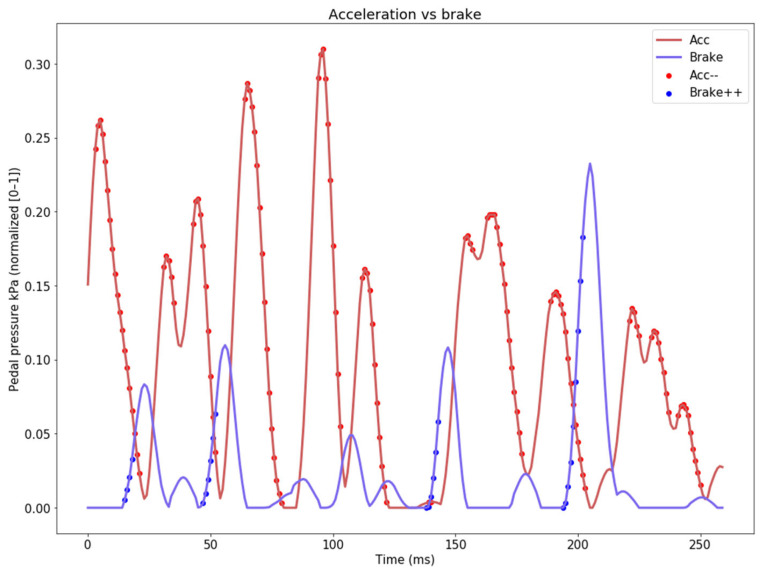
Visualization of the first-order derivative of the ascending brake signal and the descending acceleration signal.

We decided on using the ascending slope (gradient/first-order derivative) of the brake signal to detect hard braking (we also analyzed the descending slope of the acceleration, but it is not used—illustrated in Figure 4).

**Figure 5:**
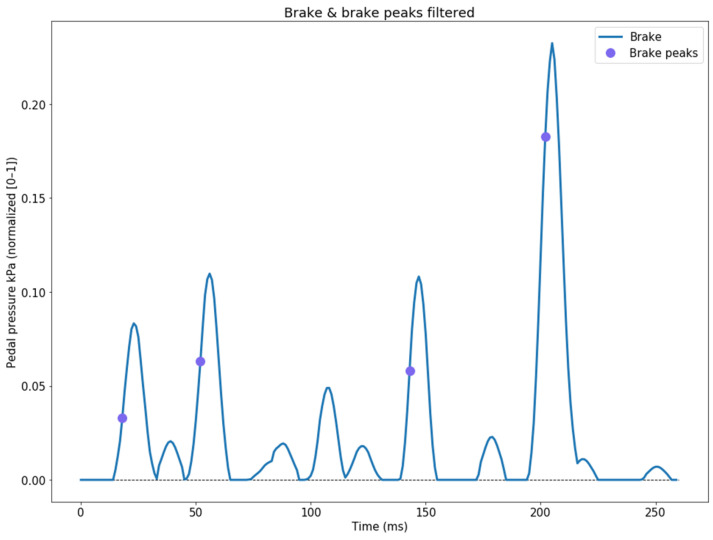
Processing the data obtained from the first-order derivative of the ascending brake signal.

We subsequently filter the peak values from the first-order derivative of the ascending brake signal (Figure 5).

**Figure 6:**
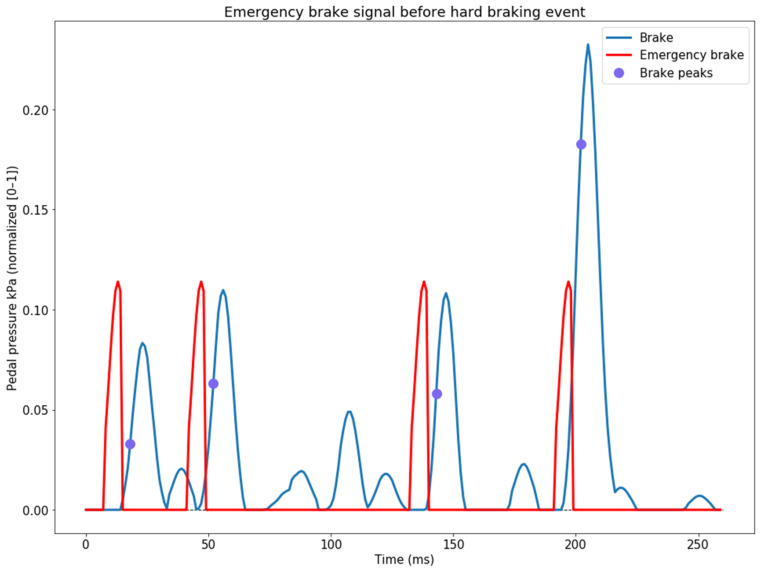
The generated emergency-brake signal modelled as a Gaussian distribution, inserted one frame before the detected hard-braking event.

The next step refers to generating the emergency-brake signal. The initial creation of the emergency-brake signal involved modeling it as a Gaussian distribution (with a standard deviation of 10 and variance of 3.5), from which only the first half (five elements) was extracted and inserted one frame before of the detected hard-brake event (Figure 6).

**Figure 7:**
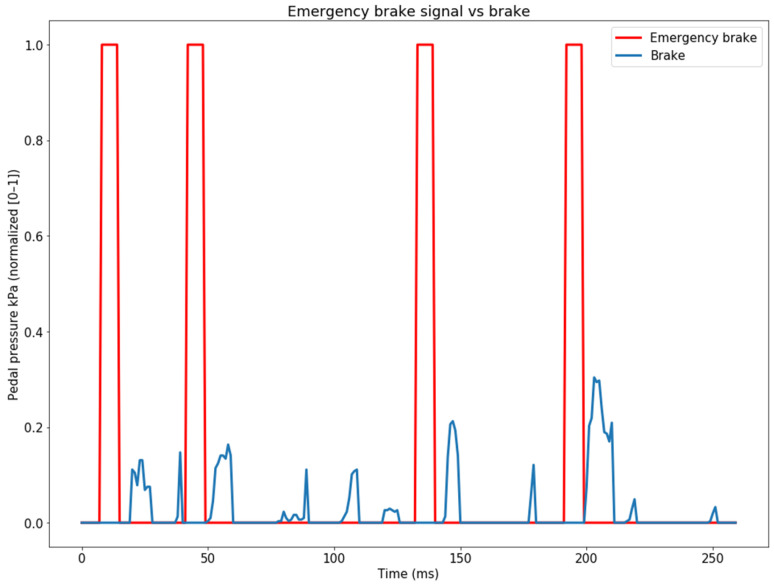
The final generated emergency-brake signal (red) and the initial brake-pedal pressure signal (blue).

Subsequently, for the purpose of optimal CNN training, we determined that converting the generated emergency signal into a step signal would be more appropriate. The generated signal is illustrated in Figure 7.

**Figure 8:**
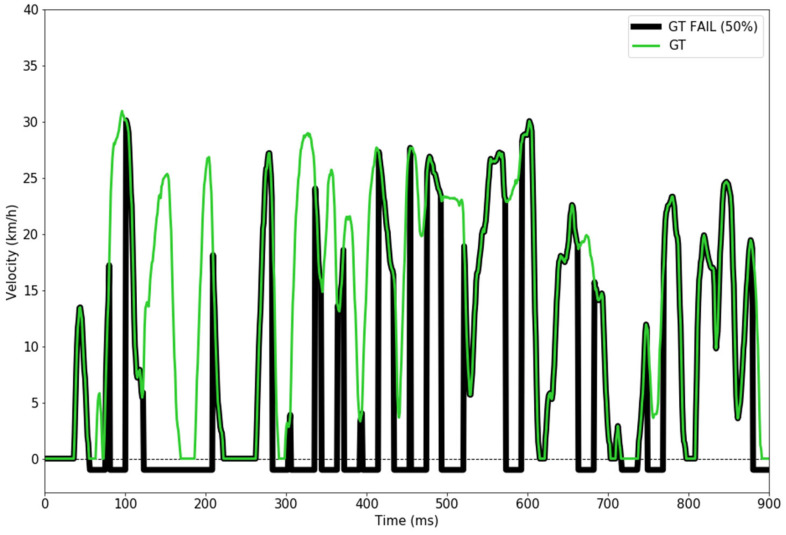
Velocity sensor with 50% failure rate vs. ground truth velocity sensor.

To ensure a robust model, we have simulated a velocity sensor failure, meaning that we randomly chose 50% of the ground truth velocity values to be represented as −1 km/h (indicating a failure in reading the velocity data) in the dataset that was used during the training phase. An example is illustrated in Figure 8.

For the evaluation of the model (described in Section 4), we have also generated, in a similar manner, failure rates for the following percentages: 10%, 20%, 30%, 50%, 80%, and 100%, that were used as velocity inputs to the CNN in order to generate the predictions.

### 3.5. Experimental Setup

The research experiments were conducted using a computational setup consisting of a desktop computer equipped with an Intel CPU with SSD storage and 64 GB RAM, and two Nvidia 1080 Ti GPUs, collectively providing a total of 22 GB VRAM during the CNN training process. The dataset employed in this research was stored on the system’s SSD storage and was accessed as needed during the training phase to effectively manage the memory limitations of the desktop workstation.

The software development environment employed for this research included Python 3.6, TensorFlow 2.12.0 [25], and Keras [26] for neural network development. Additionally, tools such as OpenCV and Matplotlib were utilized for result visualization, graph and plot generation, and the creation of video-based results.

### 3.6. Neural Network Training

**Figure 9:**
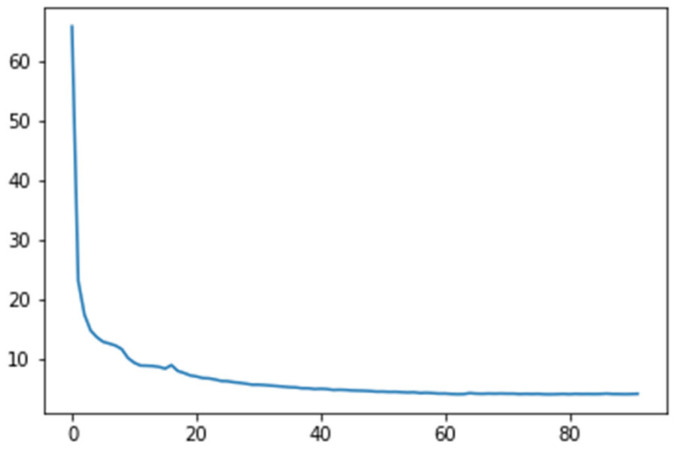
Loss function illustrated.

The CNN model we presented was trained using the Mean Squared Error (MSE) loss function, which measures the disparity between the actual ground truth values and the predicted values. The MSE loss function works by calculating the square of the differences between these values, thus penalizing larger deviations more significantly. This robustness to errors ensures that the CNN optimizes its parameters to minimize discrepancies between its predictions and the actual targets. It provides a clear and interpretable measure of the network’s accuracy. During training, we have used the Adam optimizer [27], initializing it with a learning rate of 0.001 and a decay of 0.0001. This is useful for rapid convergence during the initial training stages and fine-tuning during later epochs. The learning rate decay, or scheduling, helps prevent overshooting in the optimization process, ensuring that the model continues to learn effectively as training progresses. The evolution of the MSE loss function during training is presented in Figure 9.

The CNN was trained initially to predict velocity from raw images; then, we used those weights and retrained it to predict the additional outputs: brake-pedal pressure and emergency-brake signals. During this retraining phase, we have also used the simulated failure of the input velocity signal (50% failure rate—described in Section 3.4).

## 4. Evaluation and Results

In the evaluation section of our research paper, we employ a set of well-established metrics to assess the performance and accuracy of our model. The Mean Absolute Error (MAE) function quantifies the typical magnitude of errors between the predicted and actual values, the Root Mean Squared Error (RMSE) function provides an intuitive measure of error in the same units as the data, and the R-squared (R^2^) function measures the goodness of fit of our model. These metrics are essential for understanding and presenting our results and for evaluating the effectiveness of our approaches.

**Figure 10:**
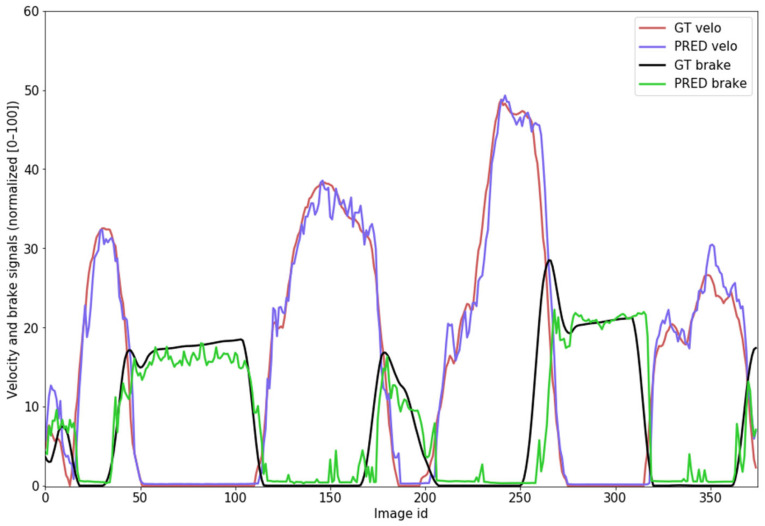
Example of predicting the current vehicle velocity and brake signals on a continuous trip (2400 total frames, only 375 plotted in the graph).

In the following part of this Section, we will present examples of predictions and evaluation results from our model. An example of the prediction of vehicle speed (velocity) and brake-pedal pressure is illustrated in Figure 10.

Results of the end-to-end model on multiple trips are presented in Table 1.

**Figure 11:**
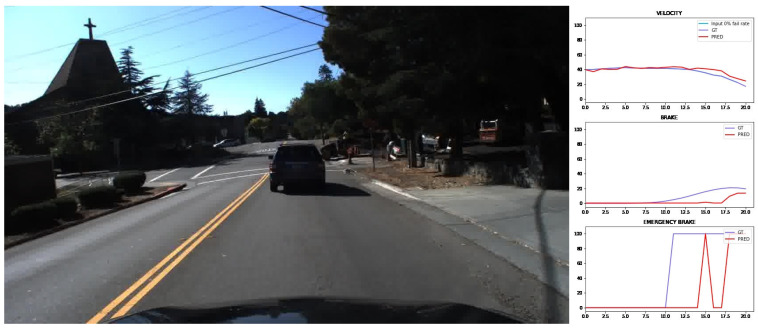
Prediction example with vehicle in front. Ground truth illustrated with purple and prediction with red.

**Figure 12:**
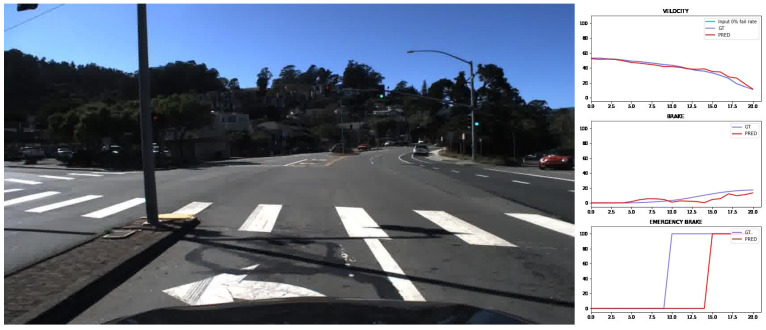
Prediction example in an intersection. Ground truth illustrated with purple and prediction with red.

Prediction examples for the three outputs of the CNN and the road-traffic image from evaluation trips are represented in Figure 11 and Figure 12.

Videos of our results on trips from the evaluation set can be accessed here: https://vimeo.com/878237978 (accessed on 7 November 2023). and https://vimeo.com/878240941 (accessed on 7 November 2023).

**Figure 13:**
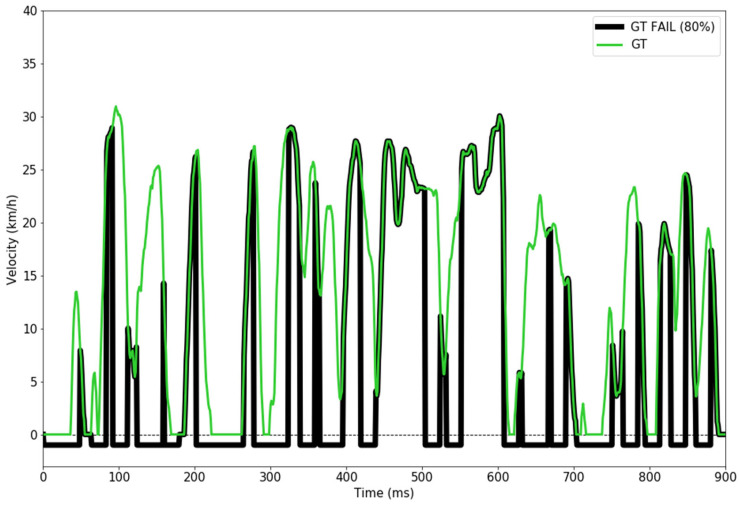
Example of simulated velocity sensor fail rate (80% of the entire trip) versus original ground truth velocity that was used to evaluate the CNN model. The values are expressed in km/h and the velocity sensor failure is set to −1 km/h.

To gain a proper understanding of the results and to test the robustness of the model’s predictions, we have tested using a velocity input as a simulated sensor failure, meaning that the input of the CNN can also be −1 km/h. We tested with various percentages of failed sensorial input for the velocity as described in Section 3.4. Figure 13 presents a simulated input of an 80% fail rate of the velocity input that was tested.

A detailed evaluation for each predicted signal on trip “201703081617” from the HDD is presented below.

### 4.1. Vehicle Velocity

The ego-vehicle velocity values were initially expressed in km/h, ranging from 0 km/h to a maximum of 137 km/h in the dataset, but the velocity data is normalized between the 0–100 interval for training and evaluation. The evaluation of the vehicle velocity is presented in Table 2.

**Figure 14:**
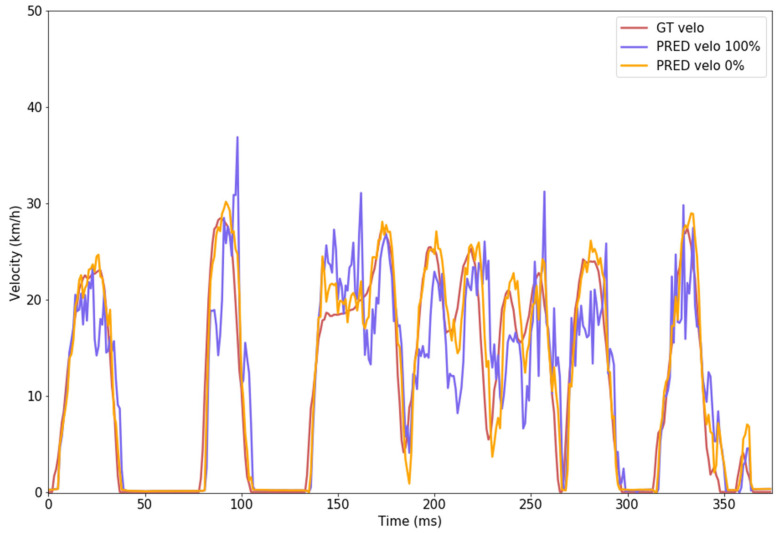
Visual representation of the predicted velocity using the actual velocity as input (0% fail rate of the sensor—yellow) versus full sensor failure (100% fail rate—purple). The actual ground truth value for the ego-vehicle velocity is represented with red.

Regarding the sensor failure used as input, in Figure 14 we present comparative results when predicting on the same data with a 0% fail rate input versus a 100% fail rate input (−1 km/h fed into the CNN).

### 4.2. Brake-Pedal Pressure

**Figure 15:**
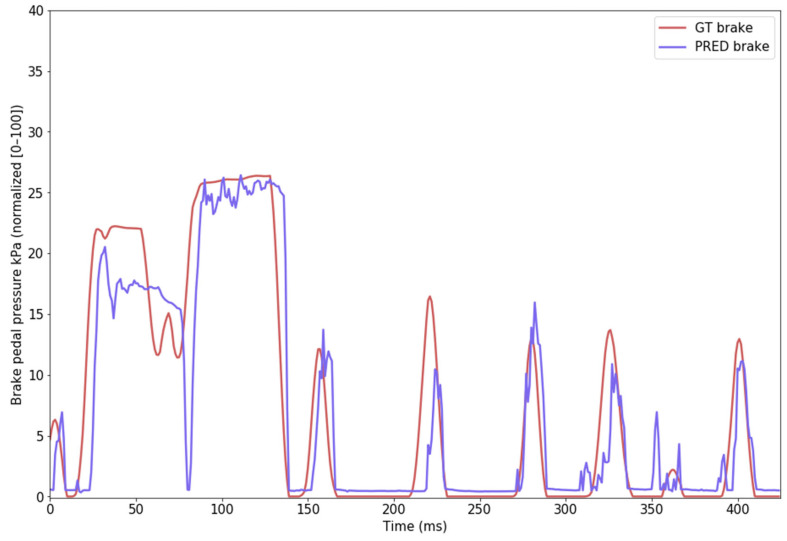
Brake-pedal pressure example of prediction (purple) vs. ground truth (red).

Values for the brake-pedal pressure signal were initially expressed in kPa, ranging from 0 kPa (meaning that no brake pressure is applied, brake pedal is released) to ~7300 kPa (brake pedal pressed to the maximum) in the dataset, but the data is normalized between the 0–100 interval for training and evaluation. The evaluation of the brake-pedal pressure is presented in Table 3 and prediction examples are illustrated in Figure 15.

### 4.3. Emergency-Brake Signal

Values for the emergency-brake signal are normalized between the 0 (no emergency brake needed) and 100 (full emergency brake required) interval for training and evaluation. Table 4 presents the evaluation of the emergency-brake signal.

Examining the R-squared results could indicate that the model does not accurately predict the expected output. However, the emergency-brake signal can be better evaluated using binary classification metrics, such as: True Positive, True Negative, False Positive, False Negative.

Therefore, to better evaluate the need for an emergency-brake event, we have computed the following:

- True Positives (TP): these are the instances where the model correctly predicts the presence of emergency-brake signals.
- True Negatives (TN): these represent the cases where the model accurately predicts the absence of emergency-brake signals.
- False Positives (FP): these are cases where the model incorrectly predicts emergency-brake signals when none were actually present.
- False Negatives (FN): these are instances where the model fails to predict the occurrence of an emergency-brake signal when one is, in fact, present.

**Figure 16:**
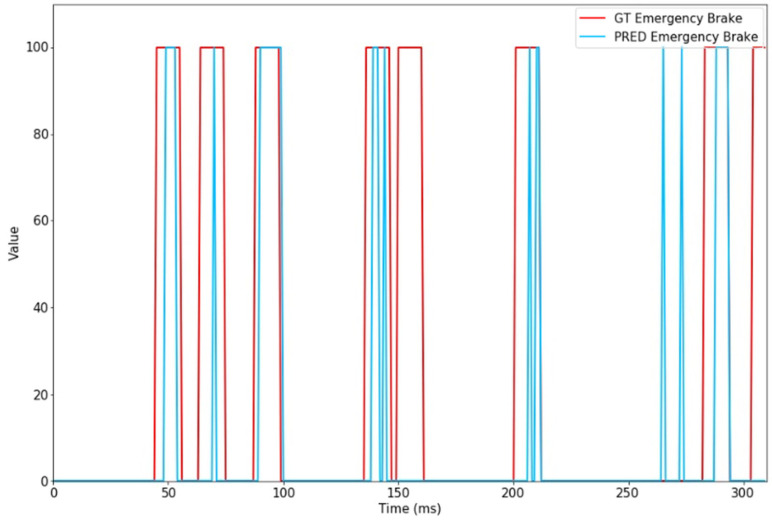
Example of emergency-brake prediction (blue) vs. ground truth (red).

As we analyze sequences of images, which constitute continuous data, we can identify a significant emergency-brake event by examining a 10-frame window. In this context, when computing false positives, we included all instances of emergency-brake signals lasting at least 10 frames. For computing the true positives, any signal lasting more than a single frame was considered. Additionally, we have also computed the True Positive Rate (Recall), False Positive Rate, Precision, and F1-Score. The results from the evaluation dataset are presented in Table 5 and prediction examples in Figure 16.

### 4.4. Compare Velocity Prediction Using a LSTM-Based Method

We have also implemented our own version of a CNN + LSTM model, based on previously published work. The model we implemented features the same input: pairs of 20 images and 20 corresponding velocity values. The layer structure of this model is similar to one published by the authors of [16], with the minimal changes required for our measurement model input. The output of the CNN + LSTM network represents the prediction of the current velocity. A comparison of this method with our own implementation is presented in Table 6.

An evaluation of the velocity prediction, on the trip ID “201709221313” and “201709221435” from the dataset, is displayed in Table 7, where we present the results obtained on the CNN + LSTM-based model against our own CNN-based model.

Although the results of the CNN + LSTM-based method are better, the model fails when tested with the simulated sensor failure. The evaluation with the various failure rates of the velocity on trip “201709221313” is presented in Table 8.

From Table 8, we can conclude that the proposed model is more robust to sensor failure and the results are better once the failure rate of the input velocity data exceeds 10%.

## 5. Conclusions

In this research, we introduce a convolutional neural network (CNN) model designed to provide accurate predictions for several critical aspects, including the current velocity of the ego-vehicle, the necessity for braking, and the demand for emergency braking, achieved through the generation of three distinct output signals. This accomplishment is made possible by making use of an end-to-end CNN architecture, which extracts visual information from images depicting road-traffic scenes alongside ego-vehicle velocity data.

Our research demonstrates the model’s robustness in handling input errors that may arise due to malfunctioning velocity sensors. Notably, our approach stands apart from the conventional methods of brake detection and speed estimation, as it does not rely on recurrent neural networks or long short-term memory networks that store historical data, streamlining the implementation and deployment processes.

In summary, the approach we propose features a reduced parameter count compared to similar prior studies, while offering a performance that is at least on par with, if not superior to, existing methodologies.

The suggested solution is applicable in real-time systems, utilizing just a single camera to anticipate potentially dangerous scenarios while driving in urban or highway settings, and holds potential to be used as a forward collision-warning (FCW) system. Furthermore, beyond its initial application, the research’s impact extends to the domain of video forensics and accident analysis. We have evaluated our proposed model using established performance metrics on publicly accessible datasets.

The limitations of the proposed method include the potential challenges in handling data variability, depending on the quality of the training data. To address these limitations, future work could focus on augmenting datasets to encompass diverse scenarios. Additional future work will involve integrating these results with a tracker, in order to better estimate the actions required for the ego-vehicle, and potentially to analyze the behavior of the other road participants. Additionally, we will explore further prediction outputs for the CNN model, including aspects such as optical flow.

## Figures and Tables

**Table 1:** Initial evaluation for the predicted vehicle state signals (velocity, brake, and emergency brake) on various sequences/trips from the used dataset.

Trip ID	Evaluation Metric	Velocity	Brake	Emergency Brake
201703081617	MAE	1.621	2.030	1.567
	RMSE	2.260	3.356	2.881
201709201605	MAE	1.602	2.372	1.737
	RMSE	2.458	4.024	3.105
201709221313	MAE	1.609	2.698	1.799
	RMSE	2.375	4.678	3.246
201709221435	MAE	1.556	2.257	1.543
	RMSE	2.224	3.786	2.963
201709221527	MAE	1.397	1.762	1.593
	RMSE	2.031	3.525	3.114

**Table 2:** Evaluation of the velocity signal using regression metrics.

Sensor Failure Rate	R-Squared	MAE	RMSE
0%	0.953	1.621	2.260
10%	0.894	2.042	3.360
20%	0.849	2.340	4.053
30%	0.826	2.657	4.340
50%	0.820	2.850	4.415
80%	0.644	3.733	5.845
100%	0.737	3.684	5.232

**Table 3:** Evaluation of the brake-pedal pressure signal using regression metrics.

Sensor Failure Rate	R-Squared	MAE	RMSE
0%	0.778	2.030	3.356
10%	0.763	2.112	3.483
20%	0.724	2.272	3.765
30%	0.721	2.319	3.792
50%	0.718	2.397	3.824
80%	0.617	2.751	4.438
100%	0.609	2.727	4.391

**Table 4:** Evaluation of the emergency-brake signal using regression metrics.

Sensor Failure Rate	R-Squared	MAE	RMSE
0%	0.274	1.567	2.881
10%	0.287	1.543	2.855
20%	0.231	1.605	2.966
30%	0.225	1.627	2.976
50%	0.187	1.692	3.048
80%	0.147	1.726	3.123
100%	0.156	1.713	3.106

**Table 5:** Emergency-brake signal evaluation.

Trip ID	201703081617	201709201605	201709221313	201709221435	201709221527
**Total frames**	**2549**	**2375**	**7443**	**5203**	**12,729**
**GT Signals (count)**	**32**	**11**	**53**	**22**	**114**
True Positive (TP)	32	11	52	22	99
False Positive (FP)	12	12	12	12	12
True Negative (TN)	2331	2352	7353	5124	12,603
False Negative (FN)	0	0	1	0	15
True Positive Rate (TPR)	1.0	1.0	0.981	1.0	0.868
False Positive Rate (FPR)	0.005	0.005	0.001	0.002	0.0009
Accuracy	0.994	0.994	0.998	0.997	0.997
Precision	0.727	0.478	0.812	0.647	0.891
F1-Score	0.842	0.647	0.888	0.785	0.880

**Table 6:** Comparison of the proposed model and a LSTM-based model.

Model	Prediction Time (ms)	Number of Parameters
CNN + LSTM	18.6	~26 million
**Proposed model**	3.1	~4.9 million

**Table 7:** Evaluation of the proposed model and a CNN + LSTM-based model.

Evaluation Set	Model	R-Squared	RMSE	MAE
201709221313	CNN + LSTM	0.998	0.838	0.583
	Proposed model	0.973	2.375	1.609
201709221435	CNN + LSTM	0.998	0.787	0.531
	Proposed model	0.984	2.224	1.556

**Table 8:** Comparing the results for velocity prediction on the same test set.

Sensor Failure Rate	R-Squared		MAE		RMSE	
	CNN + LSTM	Proposed Model	CNN + LSTM	Proposed Model	CNN + LSTM	Proposed Model
0%	0.99	0.98	0.53	1.55	0.78	2.22
10%	0.85	0.87	2.69	2.69	9.32	6.31
20%	0.52	0.86	6.02	2.77	16.78	6.42
30%	0.43	0.77	8.00	3.77	18.36	8.49
50%	0.11	0.73	11.43	4.48	22.92	9.24
80%	−0.29	0.70	16.53	5.10	27.79	9.65
100%	−1.19	0.83	26.64	4.61	36.10	7.12

## Data Availability

Data are contained within the article.
